# Management of Young Athletes with Asymptomatic Preexcitation—A Review of the Literature

**DOI:** 10.3390/diagnostics10100824

**Published:** 2020-10-15

**Authors:** Tomasz M. Książczyk, Radosław Pietrzak, Bożena Werner

**Affiliations:** Department of Paediatric Cardiology and General Paediatrics, Medical University of Warsaw, 02–091 Warsaw, Poland; tksiazczyk@wum.edu.pl (T.M.K.); radoslaw.pietrzak@wum.edu.pl (R.P.)

**Keywords:** asymptomatic preexcitation, athlete, WPW

## Abstract

Introduction: The management of young athletes with asymptomatic preexcitation remains a challenge, regardless of the progress we have made in understanding the basis of condition and developing catheter ablation procedures. The risk of sudden death, however small, yet definite, being the first symptom is determining our approach. The aim of the study was to establish the current state of knowledge regarding the management of young athletes diagnosed with asymptomatic preexcitation, by conducting a literature review. Material and methods: A comprehensive literature review was completed in accordance to the Preferred Reporting Items for Systematic Reviews and Meta-Analyses (PRISMA) guidelines. The search was limited to English language publications using the following search terms: “asymptomatic” or “incidental” and “pre-excitation” or “Wolff–Parkinson–White” or “delta wave” and “athlete” or “sport”. The search was supplemented by hand review of the bibliographies of previous relevant systematic reviews. Results: The search resulted in 85 of abstracts, and the manual search of the bibliographies resulted in 24 additional papers. After careful analysis 10 publications were included in the review. In all but one of the presented papers, the authors used non-invasive methods and then either trans-esophageal or invasive EPS as a way to risk stratify asymptomatic patients. Evidence of rapid conduction through the accessory pathway was considered high risk and prompted sport disqualification. In the analysed reports there were combined: 142 episodes of the life-threatening events (LTE)/sudden death (SCD), of which 56 were reported to occur at rest, 61 during activity and no data were available for 25. Conclusions: athletic activity may impose an increased risk of life-threatening arrhythmias in patients with asymptomatic preexcitation; hence, a separate approach could be considered, especially in patients willing to engage in high-intensity, endurance and competitive sports.

## 1. Introduction

Regardless the progress in understanding the electro-pathophysiological basis of the condition, management of asymptomatic young patients with preexcitation remains controversial. We have gone a long way, from the Wolff, Parkinson, and White landmark paper (published in 1930), through description of the mechanisms of atrio-ventricular re-entry tachycardia, linking the episodes of atrial fibrillation with risk of sudden death, to finally developing the modern technique of catheter ablation [1,2]. Further, there are still considerable gaps in our knowledge. Risk of sudden death being the first symptom determines our approach. Preparticipation screening of young athletes is becoming a standard procedure in many countries, leading to increasing numbers of detected asymptomatic patients with ventricular preexcitation (VPE) pattern on the surface electrocardiogram (ECG), who are referred to a cardiologist for risk stratification and treatment or clearance for sport activity [3]. Young athletes are generally at higher risk of arrhythmic events, and the question arises whether they need a separate approach with regard to preexcitation [4,5]. There have been a number of studies published in recent years that tried to estimate the risk of life-threatening events in asymptomatic preexcitation and the usefulness of both invasive and non-invasive tools for its prediction. However, the majority of the major studies on the subject do not address the question of risk related to sport activity. The aim of this study is to establish the risks and management with regard to physical activity and competitive sport in patients with asymptomatic preexcitation.

## 2. Materials and Methods

### 2.1. Search Strategy

**Figure 1:**
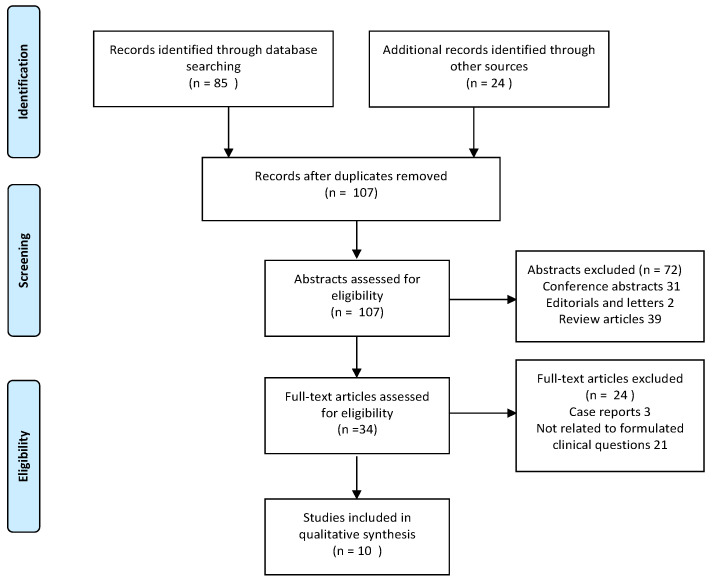
Flowchart detailing the selection of studies for the analysis.

The authors conducted a comprehensive search for all types of studies in PubMed and EMBASE using the Preferred Reporting Items for Systematic Reviews and Meta-Analyses (PRISMA) checklist [6]. The search was performed through May 2020 and limited to English language publications published between the years 1990 and 2020, using the following search terms: “asymptomatic” or “incidental” and “pre-excitation” or “Wolff–Parkinson–White” or “delta wave” and “athlete” or “sport”. The search was supplemented by hand a review of the bibliographies of previous relevant systematic reviews (Figure 1).

### 2.2. Selection Criteria

Articles were selected following the title and abstract review on the basis of their relevance to the investigated subject. We formulated following the clinical questions:

1. What was the management of young asymptomatic athletes with VPE in the published studies, and how were they stratified to risk categories?
2. Are the patients with VPE at higher risk of life-threatening arrhythmias while performing physical activity?
3. Should young athletes with VPE be managed differently than other young asymptomatic patients with VPE?

Studies were included if they reflected on the questions formulated above and provided information regarding non-invasive and invasive testing of the properties of the accessory pathway, criteria used for determining high risk patients, and incidence of the life-threatening events (LTE) or sudden death (SCD) including aborted SCD, in the context of the type of activity at the onset of symptoms.

## 3. Results

The search resulted in 85 abstracts that were reviewed and evaluated by two authors. Manual search of the bibliographies resulted in 24 additional papers. After careful analysis 10 publications were found to provide data relevant to the clinical questions formulated initially.

### 3.1. Management of Young Athletes with Preexcitation

The details of the analysed studies are summarised in Table 1.

In 2003, Sarubbi et al. [8] followed 98 asymptomatic patients for a mean of 48 months. The mean age at the recruitment was 9.7 years (+/− 5.4). Patients were assessed non-invasively at recruitment with clinical examination, ECG, echocardiogram, ECG Holter, and, when possible, exercise testing. Invasive EPS was offered to all the patients. During the follow-up five patients turned symptomatic with supraventricular tachycardia (SVT), and there was one sudden death with no remark about the circumstances. Patients with inducible arrhythmia were offered either medical treatment or RF ablation. In patients with no SVT inducible but with 1:1 conduction over the pathway ≤250 ms in the control state or 220 ms after isoproterenol were discharged without treatment but prohibited from participating in sport activities. The authors comment that this could be seen as controversial but, in their opinion, it was necessary to prevent sudden death.

In 1993 Brembilla-Perrot and Ghawi [7] published a paper that described 40 asymptomatic patients age 35 +/− 15 years, who were followed for a median of 1.8 years. All underwent either invasive electrophysiology study (EPS) or transoesophageal EPS. Patients were considered high risk if the shortest RR interval between pre-excited beats was measured as <250 ms in the control state, or <200 ms during isoproterenol infusion, also in the case of atrial vulnerability or induction of ventricular fibrillation. High-risk features were found in five patients. Two of them underwent ablation procedure, and the other three were discharged and prohibited from participation in competitive sports.

In 2007, Brembilla-Perrot et al. [9] reported a prospective study of 51 asymptomatic patients, mean age 14 years (+/− 3), with average follow-up of five years (+/− 1). All patients underwent initial assessment with ECG, 24-h Holter and exercise test, followed by a transoesophageal EPS. High-risk patients were defined if there was a rapid conduction over the pathway ≤250 ms in control and ≤200 after isoproterenol. All high-risk patients were given the recommendation to withdraw from competitive sports. Low-risk and non-inducible patients were allowed to participate. There was one aborted sudden death in a 12-year-old child with high-risk pathway, who did not have an ablation. At the onset of symptoms, the patient was running with other children. High-risk and inducible patients were offered a catheter ablation.

Fazio et al. in 2009 [10] presented a retrospective analysis of 124 children, median age 7.4 years, followed for a median of 4.2 years. Routine tests (ECG and Holter) were performed. An exercise test was performed in 76 patients. Eight children who wished to participate in competitive sports were offered a transoesophageal EPS. Two of them were found to have pathway of high-risk properties defined as 1:1 conduction over the pathway ≤210 ms or atrio-ventricular refractory period ≤230 ms. There were no LTEs recorded during the follow-up.

In 2016, Mambro et al. [11] published a report on 91 asymptomatic athletes, aged 11.8 +/− 2.28 years, with three years of follow-up. All patients underwent non-invasive assessment including 24-h ECG and exercise test. That was followed by transoesophageal EPS at rest and during exercise test and/or isoproterenol infusion. Based on the result, patients were assigned to three risk categories: low, borderline and high. High-risk was defined as having shortest pre-excited RR interval (SPERRI) ≤250 ms at rest or ≤220 ms during physical stress or sustained atrio-ventricular reentry tachycardia (AVRT) inducibility. Borderline risk was defined as inducibility of non-sustained AVRT and/or an accessory pathway effective refractory period (APERP) of 250 +/− 5 ms at rest, and/or an APERP of 220 +/− 5 ms during physical stress or isoproterenol infusion. Low risk patients were cleared for sport participation. High-risk patients were referred for catheter ablation and were allowed to participate only if the procedure. Patients with unsuccessful attempts or those who refused to have ablation were considered non-eligible for competitive sports. Borderline patients were offered ablation but not as a requirement for sport participation. There were no LTEs recorded during the follow-up period.

### 3.2. Risk of Life-Threatening Arrhythmia with Sport Activity

The details of the analysed studies are summarised in Table 2

In 1993, Munger et al. [12] attempted to examine the natural history of WPW. In a retrospective manner, they analysed 113 patients included over 36 years of follow-up. Fifty-three of those patients were asymptomatic at the time of diagnosis. There were no sudden deaths in the asymptomatic group over a cumulative period of 537 patient-years. However, 11 patients became symptomatic with tachycardias or palpitations. The patients who became symptomatic were significantly younger at the time of diagnosis than those who remained asymptomatic. Twenty-two patients from the asymptomatic group underwent EPS, and significant arrhythmias were inducible in 18 of them. In the symptomatic group there were two sudden deaths, one during athletic activity.

Important data came from a study by Timmermans et al. in 1995 [13]. A total of 690 patients were retrospectively analysed, 15 of them (mean age 28 +/− 10) suffered from out of-hospital ventricular fibrilliation (VF) and were successfully resuscitated. In eight of them, cardiac arrest was the first manifestation of the condition. At the time of onset of symptoms six patients were exercising, four were under emotional stress, two were at rest, and one was sleeping; data were not available for the two remaining patients. All patients underwent EPS, which showed that in 11 patients, the mean shortest pre-excited RR interval during induced atrial fibrillation was: 206 +/− 42 ms (range 140 to 290).

Furlanello et al. [14] in 1995 performed a retrospective analysis of 1325 competitive athletes (mean age 20.7 years) evaluated at their institution over 19 years. Among them, 380 were diagnosed with WPW. There were six patients with aborted SCD during sport activity as a presenting symptom. There were two more patients diagnosed with VPE, who refused further investigations and that had SCD while playing. Transoesophageal EPS was offered to survivors of the LTE and all patients with WPW.

An interesting analysis was provided by Finocchiaro et al. [15] in 2017. The authors of this report analysed 3684 autopsies performed for SCD and identified a subgroup of 19 patients who had diagnosis of ventricular preexcitation before death. Five of them were asymptomatic. In five cases, additional cardiac pathology was found: hypertrophic cardiomyopathy (HCM), coronary artery disease, cardiac sarcoid. In another four cases autopsy revealed findings of uncertain significance. In two patients, SCD occurred at the time of physical exercise (one in the asymptomatic group); in the remaining patients SCD happened at rest, during sleep, or no data were provided. Five patients had a history of RF ablation for WPW, which was reported to be successful in four of them.

In 2018, a retrospective, multicentre study was published by Ethridge et al. [16], which included 912 patients ≤21 years of age with diagnosed preexcitation, who underwent invasive EPS. Ninety-six patients suffered LTE (case subjects): sudden death, aborted sudden death, or atrial fibrillation with haemodynamic compromise, were compared with the control cases who had no history of LTE. In 62 patients, LTE was a presenting symptom. At the onset of LTE 43 patients were exercising (44%), among them 10 at competitive level (10%), 37 were at rest (39%), and there were no data available for 16 patients—the authors do not distinguish asymptomatic from symptomatic patients here. Accessory pathway characteristics determined during the EPS were compared between the groups. Patients with LTE were more likely to have at least one AP characteristic considered high-risk. SPERRI, APERP, and SPPCL (shortest paced cycle length with preexcitation) were significantly shorter in the case subjects; however, 37% of patients who had at least two characteristics of the AP measured, would be stratified as low-risk patients, and 25% had neither concerning AP parameters nor AVRT inducible.

## 4. Discussion

Management of young asymptomatic patients with ventricular preexcitation has always been controversial. This is due to problems with risk stratification, which is not straightforward, and, unlike in cardiomyopathies, imaging studies are not very helpful [17]. As we know, there is a small but definite risk of SCD, which is believed to be higher in younger patients and in males [18]. In the analyzed studies the age of the patients was inhomogeneous, however in all of them the mean patient age was below 40 years. The tools traditionally used to assess the risk of SCD are both non-invasive tests and EPS, all of which aim to measure the capability of the accessory pathway to rapidly conduct in antegrade fashion, as well as the inducibility and sustainability of arrhythmias. However, the reliability of those tools has been questioned, as there are problems with general anaesthesia (often used in the paediatric population) affecting the conduction system, and there is no standardisation for the use of isoproterenol [19]. Sudden death and life-threatening arrhythmias can be the first manifestation of the condition, and they have also been reported in patients with intermittent preexcitation and properties of the AP believed to be benign [16,20,21,22]. In the literature, there are reports with low and high frequency of LTE in asymptomatic children [23,24].

However, when it comes to managing young athletes with asymptomatic pre-excitation, the analyzed studies presented similar approaches. Evidence of the high-risk features of the AP usually prompts disqualification from competitive activities. In all but one of the presented papers the authors used non-invasive methods, but then they used either transoesophageal or invasive EPS as a way to risk stratify asymptomatic patients. The definition of high-risk pathway that has been used by all authors—i.e., evidence of rapid conduction over the pathway with either induced atrial fibrillation or atrial pacing manoeuvres - was proposed for the first time in 1979 by Klein et al. [2]. Shortest pre-excited RR interval (SPERRI) was suggested as marker of high-risk pathway; in later studies other parameters like APERP were proposed as useful [24]. Other recognised risk factors for SCD are younger age, multiple pathways, inducibility of the AVRT during EPS, and septal location of the accessory pathways [25,26,27].

In the majority of analysed studies, isoproterenol was used to enhance the AP properties and mimic the state of physical activity for risk stratification. Regardless of the small differences in determining the exact threshold for identifying a high-risk pathway, the authors of all the reports share the belief that the presence of the high-risk features of the pathway is enough to prohibit an asymptomatic athletes from participating in sport activities. Importantly, most of them were depending on the either transoesophageal or invasive EPS rather than on non-invasive assessment alone. Some of the authors make the distinction between competitive sport and general physical activity performed in a school environment.

This shows there is a conviction among the researchers that physical activity predisposes to the occurrence of LTE in the presence of the accessory pathway. Sport participation was retained only upon successful ablation of the accessory pathway.

This approach is also reflected in the international guidelines. ESC recommendations for competitive sport participation published in 2005 mandate that risk stratification for asymptomatic athletes is performed with EPS, and RF ablation should be performed in patients with high-risk properties of the AP. Successful ablation or the absence of the risk criteria is a prerequisite for sport eligibility [28].

Published in 2006, ESC Recommendations for leisure-time physical activity suggests that initial assessment could be done with non-invasive tests, reserving EPS for cases with persistent preexcitation. Similarly, to competitive sports recommendations, RF ablation is mandatory if high-risk criteria are present. In the update of those guidelines published in 2020, the same approach is presented, initial non-invasive assessment is suggested for recreational athletes, and EPS should be offered for persistent preexcitation and competitive sports above the age of 12 years [4,26].

In the most recent ESC Guidelines for the Management of Patients with Supraventricular Tachycardia from 2019 [25] and similarly in the ESC Guidelines on Sports Cardiology and Exercise in Patients with Cardiovascular Disease form 2020 it is recommended that all patients with asymptomatic VPE, who are willing to participate in competitive sports undergo invasive EPS for risk stratification and ablation if high-risk properties of the pathway are present. For recreational sport activity, the assessment can be started with non-invasive tests. It is noted that in children younger than 12 years old the risk of a fatal event is small and a conservative approach is suggested [27].

More importance is given to the non-invasive tests in the American guidelines. The PACES/HRS Consensus Statement, published in 2012 makes no distinction between asymptomatic athletes and non-athletes and recommends the same risk stratification protocol regardless of sport activity. Evidence of intermittent pre-excitation or sudden loss of delta wave are considered low risk. EPS and ablation are offered only to asymptomatic patients with high-risk features of the accessory pathway [29].

Similarly, the 2015 AHA/ACC recommendations for competitive sports eligibility conclude that: “in athletes with asymptomatic preexcitation, it is reasonable to attempt risk stratification with stress testing to determine whether the preexcitation abruptly terminates at low heart rates. If low risk is unclear, it is reasonable to recommend invasive electrophysiological evaluation, with ablation of the bypass tract if it is deemed high risk for SCD because of a refractory period ≤250 ms” [30].

Interestingly, the belief that athletic activity should be prohibited in patients with asymptomatic VPE is based on a limited number of cases. Among vast literature on preexcitation and risk of SCD, only a few reports comment on the circumstances and type of activity at the onset of symptoms. Our review identified only three studies [13,14,16] that provide data on larger groups than single cases, and one post-mortem analysis [15].

Historically, in this regard the greatest influence was made by Timmermans et al. [13], who emphasised the factors that increase the adrenergic tone, like sport activity and emotional stress, as increasing the risk of LTE. However, in this report only six patients out of 15 were exercising at the time of LTE, and the authors interpret them together with the group who suffered VF at the time of emotional stress—four patients, which according to the authors suggests, that the majority of events are provoked by adrenergic stimulation.

In the most recent data provided by Ethridge et al. [16] the total number of events is much greater and a large proportion of them were not related to exercise. Moreover, the authors distinguish between competitive and non-competitive activity, and only 10% of events could be attributed to competitive sports. In view of this, the authors conclude that competitive sport restrictions would not keep the majority of patients safe. At the same time, they would not recommend unrestricted sport participation for athletes with asymptomatic preexcitation. In addition, in this report, the authors suggest that although many of the LTEs were not related to exercise, the time spent performing physical activity is much shorter that the time spent at rest, and in this light the number of LTEs during athletic activity is disproportionally high.

Physical activity, especially on a competitive level, has been linked with increased risk of arrhythmic events in patients with pre-excitation [13,31]. However, it is difficult to establish how many sudden deaths in young athletes occur because of preexcitation, because we do not know the exact number of asymptomatic athletes with WPW pattern, nor how many of them died [32]. Sudden death during exercise is generally more common among the younger population and males [33]. In the long-term registry of SCD among young athletes in the USA, WPW was found to be responsible for about 2% of deaths due to cardiovascular reasons [34].

The rationale behind SCD in pre-excited patients is based on reports mentioned before, with a significant number of patients exercising at the onset of symptoms. At the same time, SCD has been reported in similar numbers to occur at rest and even during sleep. It is also important to consider the mechanism of SCD in pre-excited patients, which is thought to be atrial fibrillation or flutter (occurring either spontaneously or as degeneration of the AVRT) being rapidly conducted to ventricles over the accessory pathway and causing VF [2]. It has been proven that competitive athletes especially in endurance sports are at higher risk of developing atrial fibrillation, even after discontinuation of athletic activity [4,5]. On the other hand intensive training does not seem to affect the properties of the pathway itself [35].

Because of the uncertainty that comes with risk stratification of asymptomatic patients with preexcitation using available methods, and the low complication rate of ablation in the modern era, there is currently a low threshold to offer EPS and ablation to those patients [36,37]. If we agree that physical activity, especially at a competitive level, imposes an increased risk of triggering a potentially life-threatening arrhythmia, then there is even more argument to take this approach in the management of young athletes.

## 5. Conclusions

Answering the clinical questions formulated initially:

1. In the analyzed studies and in line with recommendations of the major cardiology societies (ESC, AHA/ACC, PACES/HRS), patients should be stratified into risk categories using non-invasive and invasive tests (transoesophageal EPS and invasive EPS) aimed at assessing the properties of the AP. Evidence of the rapid conduction over the pathway either with AF or pacing manoeuvres resulted disqualification from sport until successful ablation could be achieved.
2. Athletic activity may impose an increased risk of life-threatening arrhythmias in patients with asymptomatic preexcitation. However, the data are based on small numbers.
3. Currently there is a low threshold for offering ablation to asymptomatic individuals generally; however, a separate approach to asymptomatic athletes could be considered, especially in patients willing to engage in high-intensity, endurance, and competitive sports.

## Figures and Tables

**Table 1:** Details of the all analysed studies are presented here.

Study	Study Year	Author	Type of Study	Cases	Age of Pts	No of LTE/SCD
1	1993	Munger	Retrospective	53	33 +/− 16	2
2	1993	Brembilla–Perrot	Prospective	40	35 +/− 15	0
3	1995	Timmermans	Retrospective	690	28 +/− 10	15
4	1995	Furlanello	Retrospective	380	20.7	8
5	2003	Sarrubi	Retrospective	57	9.7 +/− 5.4	1
6	2007	Brembilla-Perrot	Prospective	55	14 +/− 3	1
7	2009	Fazio	Retrospective	124	7.8	0
8	2016	Mambro	Prospective	91	11.8 +/− 2.28	0
9	2017	Finocchiaro	Retrospective	19	31 +/− 15	19
10	2018	Ethridge	Retrospective	912	9.7 (+/− 5.3)	96

**Table 2:** Studies that revealed relation of life-threatening events (LTE)/sudden death (SCD) to physical activity are shown here.

Study	Study Year	Author	No of LTE/SCD	LTE/SCD at Rest	LTE/SCD at Physical Activity	No Data
1	1993	Munger	2	0	1	1
2	1995	Timmermans	15	7	6	2
3	1995	Furlanello	8	0	8 (competitive)	
4	2003	Sarrubi	1	no data	no data	1
5	2007	Brembilla–Perrot	1	0	1	
6	2017	Finocchiuro	19	12	2	5
7	2018	Ethridge	96	37	43 (10 competitive)	16
